# Supplementary material for: An Update on Apathy in Alzheimer’s Disease
Source: Geriatrics (Basel). 2023 Jul 14;8(4):75. doi: 10.3390/geriatrics8040075 (PMC10366907; doi:10.3390/geriatrics8040075)
Supplement: Supplementary file 1 [file geriatrics-08-00075-s001.zip › geriatrics-2455480-supplementary.pdf]

Supplementary

Table S1: Instruments and scales used to assess apathy

Rating Scales/Instruments to assess Apathy	
**Name of Scale/Instrument**	**Components**
Neuro-Psychiatric Inventory-Apathy (NPI-Apathy) [28,30]	Designed for use in patients with dementia (multiple aetiologies)<br>Score is based on responses from an informed caregiver, preferably one living with the patient<br>The person should have spent at least 4 h per day at least 4 days per week with the patient and who is knowledgeable about the patient's daytime and night-time behaviours<br><br>The NPI rates frequency, severity and caregiver distress at symptoms<br><br>Frequency is rated as:<br>1. Rarely—less than once per week<br>2. Sometimes—about once per week<br>3. Often—several times per week but less than every day<br>4. Very often—once or more per day<br><br>Severity is rated as:<br>1. Mild—produces little distress in the patient<br>2. Moderate—more disturbing to the patient but can be redirected by the caregiver<br>3. Severe—very disturbing to the patient and difficult to redirect<br><br>Distress is scored as:<br>0. Not at all<br>1. Minimally (almost no change in work routine)<br>2. Mildly (some change in work routine but little time rebudgeting required)<br>3. Moderately (disrupts work routine, requires time rebudgeting)<br>4. Severely (disruptive, upsetting to staff and other residents, major time infringement)<br>5. Very Severely or Extremely (very disruptive, major source of distress for staff and other residents, requires time usually devoted to other residents or activities)<br><br>NPI-Apathy includes 3 screening questions and 8 sub-questions including items such as:<br><br>Has the patient lost interest in the world around him/her?<br>Has he/she lost interest in doing things or does he/she lack motivation for starting new activities?<br>Is the patient less enthusiastic about his/her usual interests?<br>Does the patient show any other signs that he/she doesn't care about doing new things?
Apathy Evaluation Scale–self (AES-S), clinicians (AES-C), informant (AES-I) [31,32]	Designed for use in persons with dementia (multiple aetiologies)<br>The AES-S, AES-C and AES-I all assess the same 18 items, with ratings made from 1 (not at all characteristic) to 4 (very characteristic) on 18 items, for example:

	S/he is interested in having new experiences<br>S/he is interested in learning new things<br>When something good happens, s/he gets excited<br><br>AES-C: Performed as part of a structured interview between clinician and patient, based on both verbal and non-verbal information with ratings based on the past 4 weeks<br>AES-I: Reported by informant<br>AES-S: Self-reported by patient<br><br>Scores for the AES range from 18 to 72. In the original validation study (2), the mean (standard deviation) score for 30 healthy older adults were: AES-C: 26 (+/−6); AES-I: 26 (+/−7.5); AES-S: 28 (+/−6)
Apathy Scale [37]	Includes a set of 14 questions, initially validated in patients with Parkinson's Disease:<br><br>Questions include examples such as<br>Are you concerned about your condition?<br>Do you put much effort into things?<br>Are you always looking for something to do?<br>Scoring<br>For questions 1–8, not at all = 3 points; slightly = 2; some = 1; a lot = 0<br>For questions 9–14, not at all = 0; slightly = 1; some = 2; a lot = 3
Apathy Inventory [38]	Designed for use in patients with probable AD, Parkinson's disease and MCI<br>Caregiver (AI-C)- and patient (AI-P)-version both assess:<br>Emotional blunting<br>Lack of initiative<br>Lack of interest<br><br>AI-C: If the response is positive, the frequency and gravity of the item are explored<br>How frequently do these problems arise?<br>How severe are these problems; to what extent do they disturb or handicap the patient?'<br>For each of the three questions, the maximum score (Frequency 1–4, Severity 1–3) is 12, giving a maximum total score of 36<br>AI-P: The score for each dimension is obtained by using a Likert-style scale (1–12): if the symptom is present, the subject is asked to estimate its intensity (from mild at the left-hand end of the scale, to severe at the right-hand end)
Dementia Apathy Interview and Rating (DAIR) [39]	Designed for use in patients with possible and probable AD<br>Sixteen-item scale that consists of a structured interview with a caregiver—specifically designed for persons with dementia (all types)<br>The caregiver must have seen the individual with dementia at least two times each week for the previous month<br>Include items in the scale minimally related to cognitive ability (e.g., Are there things s/he is enthusiastic about? Does s/he show interest in news about friends and relatives?)<br>Respondents must record for each behaviour whether it is a change from baseline
Dimensional Apathy Scale (DAS) [40,41]	Twenty-four-item multidimensional apathy measure undertaken by carers<br>Composed of three subscales: executive, emotional, and initiation

	Examples of<br>Executive subscale “S/he finds it difficult to keep his/her mind on things”<br>Emotional subscale “S/he is indifferent to what is going on around him/her”<br>Initiation subscale “S/he acts on things s/he has thought about during the day”<br>Created specifically to better profile apathy subtypes in people with neurodegenerative and neurological disease<br>Items are scored on a 4-point Likert scale<br>The items are summed for each subscale, with the total subscale score ranging from 0 (least apathetic) to 24 (most apathetic), with a DAS total score ranging from 0 to 72
Apathy Motivation Index (AMI) [42]	Validated in healthy community dwelling population<br>Self-administered 18-item scale<br>Participants self-rate each item on a five-point Likert scale by deciding how true that statement was based on the past two weeks of their life<br>The scale ranges from 0–4 (with 0 = ‘completely untrue’, 4 = ‘completely true’)<br>Each item is reverse-scored so that a higher rating indicates more apathy<br>Composed of six items for each of three subscales<br>Behavioural activation (goal-directed behaviour initiation, e.g., “I get things done when they need to be done, without requiring reminders from others”)<br>Social motivation (interest and engagement in social interactions, e.g., “I start conversations without being prompted”)<br>Emotional sensitivity (feeling positive and negative emotions, e.g., “I feel sad or upset when I hear bad news”)
Lille Apathy Rating Scale (LARS) [43,44]	Scale was initially validated to diagnose apathy in persons with probable Parkinson’s disease<br>Comprises 33 queries belonging to 9 domains with overall score ranges from −36 to +36 and each domain corresponding to a clinical manifestation of apathy including the following:<br>Everyday productivity<br>Interests<br>Taking initiative<br>Novelty seeking<br>Motivation- voluntary actions<br>Emotional responses<br>Concern<br>Social Life<br>Self- awareness
APADEM-NH (APAthy in DEMentia, Nursing Home) scale [45,46]	Validated in nursing home residents with dementia (unspecified type)<br>Scale undertaken by caregiver known to patient<br>26 items assessed:<br>Deficits of Thinking and Self-Generated behaviours: 13 items<br>Emotional Blunting: 7 items<br>Cognitive Inertia: 6 items<br>Each item is scored from 0 to 3<br>0: No Apathy (spontaneous emotion or action)<br>1: Mild Apathy (action or emotion needs some kind of stimulation)<br>2: Moderate Apathy (action or emotion obtained only with intense stimulation)<br>3: Severe Apathy (not responding to any intervention)

Person- Environment Apathy Rating (PEAR) scale [29]	Designed for use in persons with dementia (unspecified type)<br>Consists of two subscales, PEAR-Environment and PEAR-Apathy<br>Each subscale has six items, and each item is rated on a 1–4 scale; higher ratings indicate a better environment and greater apathy, respectively<br>The total score ranges from 6 to 24 for each subscale<br><br>PEAR-Environment subscale assesses:<br>stimulation clarity, stimulation strength, stimulation specificity, interaction involvement, physical accessibility, and environmental feedback<br>PEAR-Apathy subscale evaluates:<br>facial expression, eye contact, physical engagement, purposeful activity, verbal tone, and verbal expression that reflect the symptoms of apathy in cognitive, behavioural, and affective domains
